# The interaction between sugar-sweetened beverage consumption and emotional status with subjective cognitive decline among rural older adults: a cross-sectional study

**DOI:** 10.3389/fnut.2026.1879896

**Published:** 2026-08-12

**Authors:** Yahong Guo, Jing Shi, Xinjin Li, Cailing Yang, Weijuan Kong, Meiman Li, Yuxin Mao, Min Jia, Ruixue Liu, Yanhua Ning

**Affiliations:** 1 General Hospital of Ningxia Medical University, Yinchuan, China; 2 School of Nursing, Ningxia Medical University, Yinchuan, China

**Keywords:** negative affect, positive affect, rural older adults, subjective cognitive decline (SCD), sugar-sweetened beverage (SSB) consumption

## Abstract

**Objectives:**

This study explored the interactive and joint effects of Sugar-Sweetened Beverage (SSB) consumption, positive and negative affect with Subjective Cognitive Decline (SCD) among rural older adults, providing empirical evidence for formulating targeted strategies for the early intervention of cognitive decline in the rural older population.

**Methods:**

This cross-sectional study recruited 485 valid participants from rural areas of Ningxia, China, using a stratified cluster sampling method. Key indicators were assessed with the subjective cognitive decline questionnaire, the positive and negative affect schedule, the sugar-sweetened beverage consumption questionnaire, and sociodemographic covariates. Data analysis included independent-samples *t*-test, analysis of variance, correlation analysis, and linear regression models with the PROCESS macro in SPSS for examining interaction effects.

**Results:**

Gender, education, and self-rated health were independent associated factors of SCD among rural older adults. Positive affect was negatively correlated with SCD (β = −0.238, *P* < 0.001), while negative affect was positively correlated with SCD (β = 0.377, *P* < 0.042). There was an interactive effect between SSB consumption and positive affect on SCD (β = 0.082, *P* = 0.012). Simple slope analysis showed that higher positive affect was associated with less severe SCD among no SSB consumers, whereas the protective effect of positive affect against SCD was significantly attenuated among SSB consumers. The joint effect analysis revealed that compared with the reference group (no SSB consumption + high positive affect/no SSB consumption + high negative affect): Participants in the no SSB consumption + low positive affect group had significantly higher SCD scores (β = 0.959, *P* < 0.001). Participants in the no SSB consumption + low negative affect group had significantly lower SCD scores (β = −0.778, *P* = 0.003). The SSB consumption + low negative affect group had the lowest SCD scores, with a highly significant reduction compared with the reference group (β = −0.936, *P* < 0.001).

**Conclusion:**

The inverse association of positive affect with SCD was significantly weakened among participants with SSB consumption. Regardless of SSB consumption, low negative affect corresponded to milder SCD, suggesting possible directions that require prospective confirmation.

## 1 Introduction

Global population aging drives rising prevalence of Alzheimer’s disease (AD), bringing growing focus on late-life cognitive health (1). In China, AD affects roughly 4.8% of adults aged 60 and above over the past decade, representing a major public health challenge (2). Owing to the lack of effective treatment for AD, it has become very important to identify people with risk factors or clinical signs to implement targeted prevention (3). Subjective cognitive decline (SCD), a pre-stage of Mild Cognitive Impairment (MCI) and the initial symptom of some types of dementia, is characterized by a subjective decline in cognitive function compared to the normal condition, even though the level of objective cognitive performance is not yet compromised (1). SCD is considered an early warning signal and a significant risk factor for the onset and progression of AD, since an increasing amount of research has demonstrated that it can predict the prevalence of MCI and AD over time (4, 5). In order to delay or prevent the onset and progression of AD, early detection and intervention of modifiable risk factors for SCD are crucial for public health.

Currently, the impact of lifestyle factors, such as diet, on diseases has attracted growing attention in light of the rising incidence of numerous metabolic diseases. Numerous investigations have proven a greater association between dietary intake and cognitive function, and in particular, healthy dietary patterns play a protective role on cognitive function in middle-aged and older adults (6). Consistently, the 2015 Dietary Guidelines for Americans put forward the top recommendation that calorie and nutrient contribution to the diet should be considered for shifting to healthier food and beverage choices (7). Sugar-sweetened beverage (SSB) is the largest source of beverage calories in the United States, with a large number of consumers, and people aged 60 and above account for a higher proportion of SSB consumption (8). Long-term cohort studies and cross-sectional studies in humans have found significant associations between the consumption of added sugars, specifically SSB, and reduced cognitive function, poor memory performance, and higher risk of cognitive impairment (9, 10).

Emotional states are a significant psychological element influencing the cognitive function of older adults (11). Rural older adults are more likely to experience negative feelings like anxiety and sadness due to a number of factors, including a high empty-nest rate, a lack of social activities, and a low economic status (12). Komalasari et al. (13) found that emotional vulnerability to subjective disorders increases the risk of SCD. A study by Zhu et al. showed that rural older adults with depression are more likely to develop SCD, which may be because cortisol levels are at an abnormal level in a depressed state, leading to damage, atrophy, and neuroinflammation of the hippocampus, thereby affecting cognition (14). According to a different study, older adults who have a more optimistic outlook on aging may focus more on maintaining cognitive function, which lowers the likelihood of subjective memory deterioration (15).

Currently, the majority of research has examined the relationship between SSB consumption or emotional status and cognitive function in older adults independently. However, there are still few studies that combine the three to examine their combined effects. More crucially, the prevalence of SCD among China’s rural older adults is substantially higher than that among urban older adults in China due to a weak economic basis, a lack of medical service resources, a lack of attention to diseases, and a lack of awareness of medical treatment (14). Therefore, this study focused on an underrepresented rural older population, simultaneously integrating nutritional and psychological factors associated with SCD. Specifically, we investigated the interaction between SSB consumption and emotional status, and stratified participants into four joint exposure groups to quantify their combined effects on the severity of SCD. Collectively, the study provides new insights into the interaction between nutritional and psychological factors associated with SCD and provides preliminary epidemiological evidence that may inform future longitudinal and intervention research.

## 2 Materials and methods

### 2.1 Study design and participants

Stratified cluster sampling was adopted, with 2 counties selected from each of the 5 cities in Ningxia, and 1 administrative village chosen from each county. A total of 518 permanent elderly adults from 10 sample villages were recruited as participants. Inclusion criteria: (1) Age ≥ 60 years old; (2) Having a rural household registration and residing in rural areas for at least 6 months per year; (3) With clear consciousness and able to communicate with investigators without obstacles; (4) Voluntarily participating in this study. Exclusion criteria: (1) Suffering from neurological diseases that affect cognitive function; (2) Having mental illnesses such as schizophrenia; (3) Being severely disabled and unable to cooperate with the survey. A total of 518 questionnaires were collected, with 485 valid ones, yielding an effective response rate of 93.63%. The study protocol adhered to the principles of the Declaration of Helsinki and was approved by the Ethics Committee of Ningxia Medical University (No. 2022-G018). All participants signed a written informed consent.

### 2.2 Measures

#### 2.2.1 Subjective cognitive decline

The 9-Item subjective cognitive decline questionnaire (SCD-Q9) was used to assess SCD symptoms (16). This Chinese version, developed and validated by Hao et al., exhibited satisfactory reliability with a Cronbach’s α of 0.811 (17). This scale consists of two dimensions with a total of 9 items. Total scores range from 0 to 9, where higher scores indicate more severe SCD symptoms. In the present study, the Cronbach’s α was 0.843.

#### 2.2.2 Assessment of SSB consumption

Participants reported their intake of 8 types of sugar-sweetened beverage in the past month, including carbonated drinks, freshly squeezed fruit juices, non-freshly squeezed packaged fruit and vegetable juice drinks, packaged plant protein drinks, lactic acid bacteria drinks, formulated milk drinks, tea drinks, and milk tea. The survey included whether each type of drink was consumed and the consumption frequency in the past 1 month. This measurement tool showed good internal consistency (Cronbach’s α coefficient = 0.809). Based on previous literature (18) and considering the low proportion of high-frequency consumers among rural older adults, participants who consumed SSB at least once a month were defined as the SSB consumption population in this study.

#### 2.2.3 Positive affect and negative affect schedule (PANAS)

Positive and negative affect were measured using the short-form version of the Positive and Negative Affect Schedule (PANAS) revised by Diener et al. (19). The scale comprises 12 items, with each subscale containing 6 items. A 5-point Likert scale was adopted, and participants were required to rate the intensity of their positive affect and negative affect over the past month. The Chinese version of the scale has been validated as a reliable and valid tool for assessing individuals’ emotional states (20). In the current sample, the Cronbach’s α coefficient was 0.860 for the PA subscale and 0.880 for the NA subscale.

#### 2.2.4 Covariates

Sociodemographic data, including age, gender, occupation, education, marital status, medical insurance, disease status, self-rated health, height, and weight.

### 2.3 Data collection

Before the survey, investigators underwent standardized training to learn how to use consistent wording. They also communicated with the local officials to explain the informed consent and electronic questionnaires, the purpose and significance of the study, and obtained their support and cooperation. During the survey, investigators conducted one-on-one, interview-style surveys with rural elderly residents, remaining ready at all times to answer questions, resolve issues, and conduct on-site verification. All questionnaires were completed and collected on-site to ensure their integrity.

### 2.4 Data analysis

All analyses were performed in IBM SPSS 27.0 with the PROCESS macro for interaction testing; the significance threshold was set at *P* < 0.05. Measurement data that follow a normal distribution were expressed as the mean ± SD, while categorical data were described as percentages or proportions. Independent-samples *t*-test and one-way analysis of variance (ANOVA) were adopted for univariate analysis. Pearson correlation analysis was used to explore the correlations among variables. After testing for multicollinearity and common method bias, linear regression models were constructed, and the Process macro in SPSS was further employed to examine the interaction effects of SSB consumption and positive/negative affect on SCD among rural older adults. Multiple linear regression analysis was conducted to evaluate the differences in SCD scores among different joint exposure categories.

## 3 Results

### 3.1 Common method bias test and multicollinearity test

The Harman’s single-factor test was adopted to examine the common method bias. A total of 4 factors with eigenvalues greater than 1 were extracted, and the first factor explained 35.28% of the total variance, which was less than 40%, indicating Harman’s single-factor test did not indicate severe common method variance (21). To evaluate multicollinearity, all predictor variables were tested using multiple linear regression, with variance inflation factor (VIF) values < 5, indicating no significant multicollinearity issues.

### 3.2 Univariate analysis of factors affecting SCD among rural older adults

A total of 518 questionnaires were distributed for this study. After excluding 33 invalid questionnaires, 485 valid questionnaires were obtained, resulting in an effective response rate of 93.63%. Among the participants, there were 230 males and 255 females, with a mean age of 69.81 ± 7.13 years (Table 1).

**TABLE 1:** Univariate analysis of factors affecting SCD among rural older adults (*n* = 485).

Variables	N (%)	SCD-Q9 scores		
		Mean ± SD	*t/F*	*P*-value
Age (years)			4.797^2^	0.009
60–69	244 (50.3)	7.01 ± 1.79		
70–79	189 (40.0)	7.39 ± 1.70		
≥ 80	52 (10.7)	7.23 ± 1.73		
Gender			− 5.756^1^	< 0.001
Male	230 (47.4)	6.77 ± 1.86		
Female	255 (52.6)	7.64 ± 1.50		
Occupation			2.813^1^	0.005
Farmer	137 (28.3)	6.88 ± 1.85
Non-farm workers	348 (71.8)	7.37 ± 1.67
Education			18.558^2^	< 0.001
Primary school or lower	376 (77.5)	7.48 ± 1.61		
Middle school	87 (18.0)	6.50 ± 1.89		
Technical secondary school or above	22 (4.50)	5.98 ± 1.84
Marital status			1.91^1^	0.055
Married	390 (80.4)	7.16 ± 1.74		
Non-married	95(19.6)	7.55 ± 1.65		
Types of chronic disease			17.102^2^	< 0.001
0	108 (22.3)	6.44 ± 1.94		
1	179 (36.9)	7.29 ± 1.69		
≥ 2	198 (40.8)	7.61 ± 1.50		
Self-rated health			16.079^2^	< 0.001
Healthy	48 (9.9)	6.00 ± 2.21		
Good	97 (20.0)	6.92 ± 1.47		
Fair	239 (49.3)	7.32 ± 1.72		
Poor	101 (20.8)	7.92 ± 1.32		
BMI			1.009^2^	0.398
Underweight	23 (4.7)	7.54 ± 1.48		
Normal weight	215 (44.3)	7.32 ± 1.63		
Overweight	173 (35.7)	7.19 ± 1.75		
Obesity	74 (15.3)	6.98 ± 2.01		
SSB consumption (past month)			2.705^1^	0.007
No SSB consumption	146 (30.1)	7.55 ± 1.64		
SSB consumption	339 (69.9)	7.09 ± 1.75		

^1^*t*-test;

^2^ANOVA *F*-value.

### 3.3 Correlation analysis of SCD, positive affect and negative affect

In this study, SCD scores among older adults were negatively correlated with positive affect scores (*r* = −0.238, *P* < 0.001), and positively correlated with negative affect scores (*r* = 0.377, *P* < 0.001) (Table 2).

**TABLE 2:** Correlation analysis of SCD, positive and negative affect (*n* = 485).

Variables	Scores (x̄ ± *s*)	Positive affect	Negative affect	SCD
Positive affect	14.62 ± 6.24	1	1	1
Negative affect	18.62 ± 5.07	−0.488***		
SCD	7.23 ± 1.73	−0.238***	0.377***	

***P<0.001.

### 3.4 Interactive effects of SSB consumption and positive affect with SCD among rural older adults

Taking the score of SCD in rural older adults as the dependent variable, gender, education, types of chronic disease, and self-rated health as control variables, and SSB consumption and positive affect as independent variables, a linear regression model was constructed to analyze their interaction effects (Table 3). The results showed that the overall model fit was good (*R* = 0.428, *R*^2^ = 0.184, *F* = 11.862, *P* < 0.001). After adjusting for relevant confounding factors, gender (β = 0.369, *P* = 0.021), education (β = −0.579, *P* < 0.001), self-rated health (β = 0.293, *P* = 0.016), SSB consumption (β = −0.168, *P* = 0.006), positive affect (β = −0.167, *P* = 0.004), and the interaction term between SSB consumption and positive affect (β = 0.082, *P* = 0.012) all had significant effects on the SCD score of rural older adults (*P* < 0.05).

**TABLE 3:** Interactive effects of SSB consumption and positive affect with SCD among rural older adults (*n* = 485).

Variables	β	*SE*	*t*	*P*	95% CI
Constant	10.079	1.289	7.818	<0.001	[7.546∼12.613]
Age	0.028	0.114	0.246	0.806	[−0.196∼0.252]
Gender	0.369	0.159	2.314	0.021	[0.056∼0.682]
Occupation	−0.298	0.164	−1.814	0.070	[−0.621∼0.025]
Education	−0.579	0.147	−3.934	<0.001	[−0.869∼−0.290]
Types of chronic disease	0.186	0.133	1.402	0.161	[−0.075∼0.447]
Self-rated health	0.293	0.120	2.429	0.016	[0.056∼0.529]
SSB consumption	−0.1683	0.163	−2.745	0.006	[−2.887∼−0.478]
Positive affect	−0.167	0.058	−2.885	0.004	[−0.281∼−0.053]
SSB consumption × Positive affect	0.082	0.032	2.531	0.012	[0.018∼0.145]
*R*	0.428				
*R* ^2^	0.184				
*F*	11.862				

**FIGURE 1:**
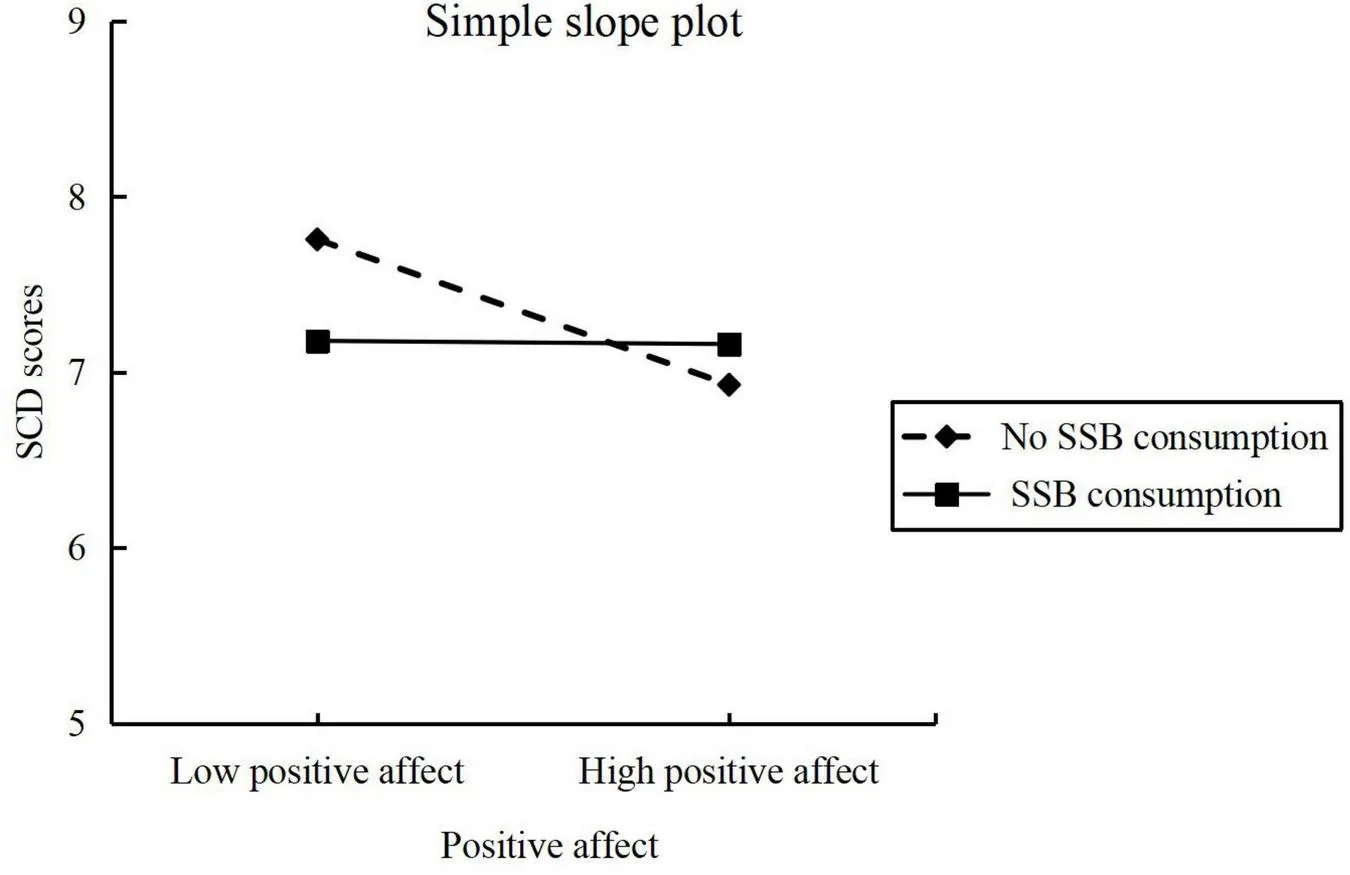
Moderating effect of SSB consumption on the association between positive affect and SCD.

To further clarify the interaction between SSB consumption and positive affect, simple slope analysis was conducted in this study (Figure 1). The results showed that among older adults who did not consume SSB, positive affect was negatively correlated with SCD scores; this negative correlation between positive affect and SCD was weaker among older adults with SSB consumption.

### 3.5 Joint effect of SSB consumption and positive affect with SCD among rural older adults

To further clarify the joint effects of SSB consumption and positive affect with SCD among rural older adults, participants were divided into four mutually exclusive joint exposure groups based on SSB consumption status (yes/no) and positive affect level (stratified into high/low groups by the median): ➀No SSB consumption + high positive affect (reference group); ➁No SSB consumption + low positive affect; ➂SSB consumption + high positive affect; ➃SSB consumption + low positive affect. Taking the total SCD score as the dependent variable, multiple linear regression was performed to analyze the differences in SCD levels across different joint exposure groups after adjusting for confounding factors such as gender, education level, and self-assessed health status, so as to quantify the joint effects of the two variables.

**FIGURE 2:**
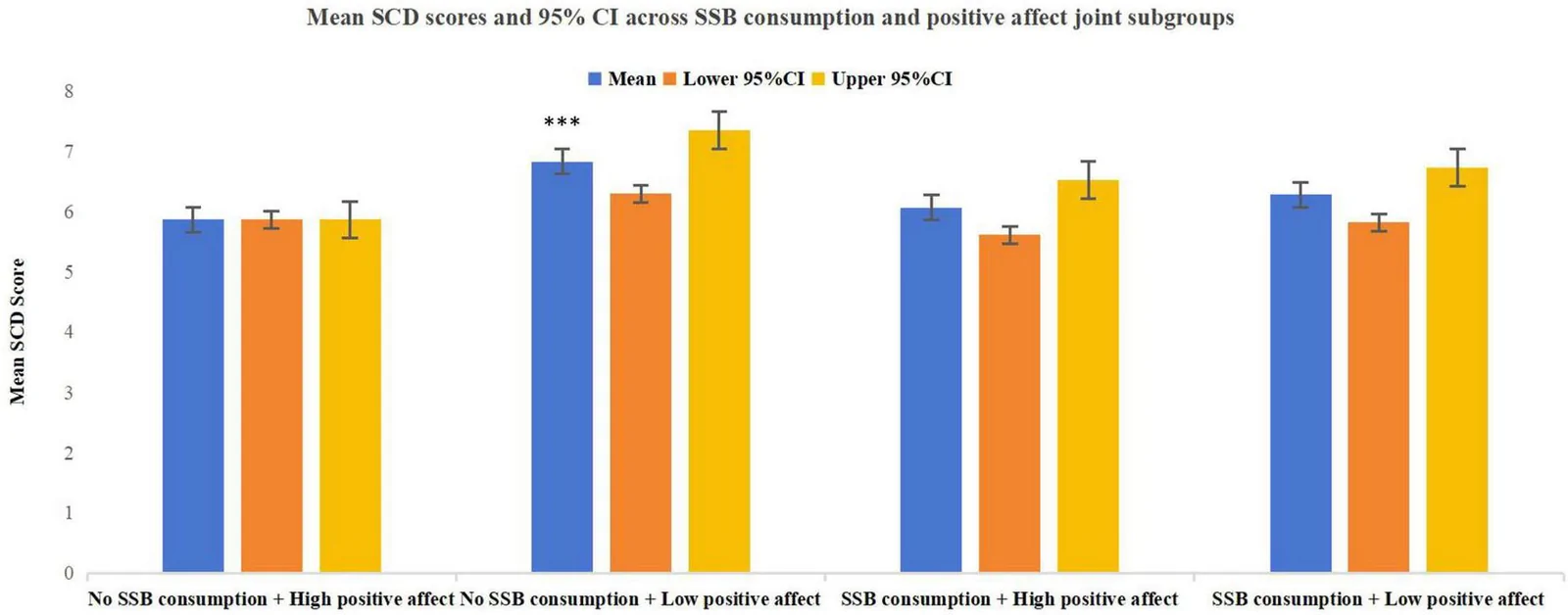
Mean SCD scores across four joint exposure groups of SSB consumption and positive affect among rural older adults. ***Statistically significant intergroup difference.

The results indicated that compared with the reference group: Participants in the no SSB consumption + low positive affect group had significantly higher SCD scores (β = 0.959, *P* < 0.001). The SSB consumption + high positive affect group showed no significant difference in SCD scores compared (β = 0.201, *P* = 0.386). The SSB consumption + low positive affect group exhibited marginally significantly higher SCD scores (β = 0.412, *P* = 0.076). The overall model fitted well (*F* = 17.792, *P* < 0.001) (Table 4 and Figure 2).

**TABLE 4:** Joint effect of SSB consumption and positive affect with SCD among rural older adults (*n* = 485).

Variables	β	*SE*	*t*	*P*	95% CI
Constant	5.870	0.440	13.355	<0.001	[5.007∼6.734]
Gender	0.388	0.157	2.467	0.014	[0.079∼0.697]
Education	−0.578	0.143	−4.049	<0.001	[−0.858∼−0.297]
Self-rated health	0.403	0.087	4.652	<0.001	[0.233∼0.573]
No SSB consumption + low positive affect	0.959	0.268	3.575	<0.001	[0.432∼1.485]
SSB consumption + high positive affect	0.201	0.232	0.868	0.386	[−0.254∼0.656]
SSB consumption + low positive affect	0.412	0.232	1.776	0.076	[−0.044∼0.867]
*R*	0.427				
*R* ^2^	0.183				
*F*	17.792***				

Statistically significant intergroup difference:

*P < 0.05;

**P < 0.01;

***P < 0.001.

### 3.6 Interactive effects of SSB consumption and negative affect with SCD among rural older adults

Taking the SCD score of rural older adults as the dependent variable, gender, education, types of chronic disease, and self-rated health as control variables, and SSB consumption and negative affect as independent variables, a linear regression model was constructed to analyze their interaction effect (Table 5). The results showed that the overall model fit was good (*R* = 0.481, *R*^2^ = 0.231, *F* = 15.841, *P* < 0.001). After adjusting for confounding factors, gender (β = 0.376, *P* = 0.016), education (β = −0.566, *P* < 0.001), self-rated health (β = 0.261, *P* = 0.025) and negative affect (β = 0.092, *P* = 0.036) had a significant positive effect on the SCD score of rural older adults, while the interaction term between negative affect and SSB consumption (β = −0.011, *P* = 0.668) showed no statistically significant effect on the SCD score (*P* > 0.05).

**TABLE 5:** Interactive effects of SSB consumption and negative affect with SCD among rural older adults (*n* = 485).

Variables	β	*SE*	*t*	*P*	95% CI
Constant	6.092	0.848	7.182	<0.001	[4.425∼7.759]
Age	0.088	0.111	0.794	0.428	[−0.130∼0.306]
Gender	0.376	0.156	2.410	0.016	[0.061∼0.554]
Occupation	−0.071	0.061	−1.177	0.239	[−0.191∼0.048]
Education	−0.566	0.142	−3.992	<0.001	[−0.845∼−0.288]
Types of chronic disease	0.141	0.129	1.093	0.275	[−0.112∼0.393]
Self-rated health	0.261	0.117	2.244	0.025	[0.033∼0.490]
SSB consumption	−0.096	0.392	−0.245	0.807	[−0.865∼0.673]
Negative affect	0.092	0.043	2.102	0.036	[0.006∼0.177]
SSB consumption × negative affect	−0.011	0.025	−0.430	0.668	[−0.059∼0.038]
*R*	0.481				
*R* ^2^	0.231				
*F*	15.841				

### 3.7 Joint effect of SSB consumption and negative affect on SCD among rural older adults.

To further clarify the joint effects of SSB consumption and negative affect on SCD among rural older adults, participants were divided into four mutually exclusive joint exposure groups based on SSB consumption status (yes/no) and negative affect level (stratified into high/low groups by the median): ➀No SSB consumption + high negative affect (reference group); ➁No SSB consumption + low negative affect; ➂SSB consumption + high negative affect; ➃SSB consumption + low negative affect. Taking the total SCD score as the dependent variable, multiple linear regression was performed to analyze the differences in SCD levels across different joint exposure groups after adjusting for confounding factors such as gender, education level, and self-assessed health status, so as to quantify the joint effects of the two variables.

**FIGURE 3:**
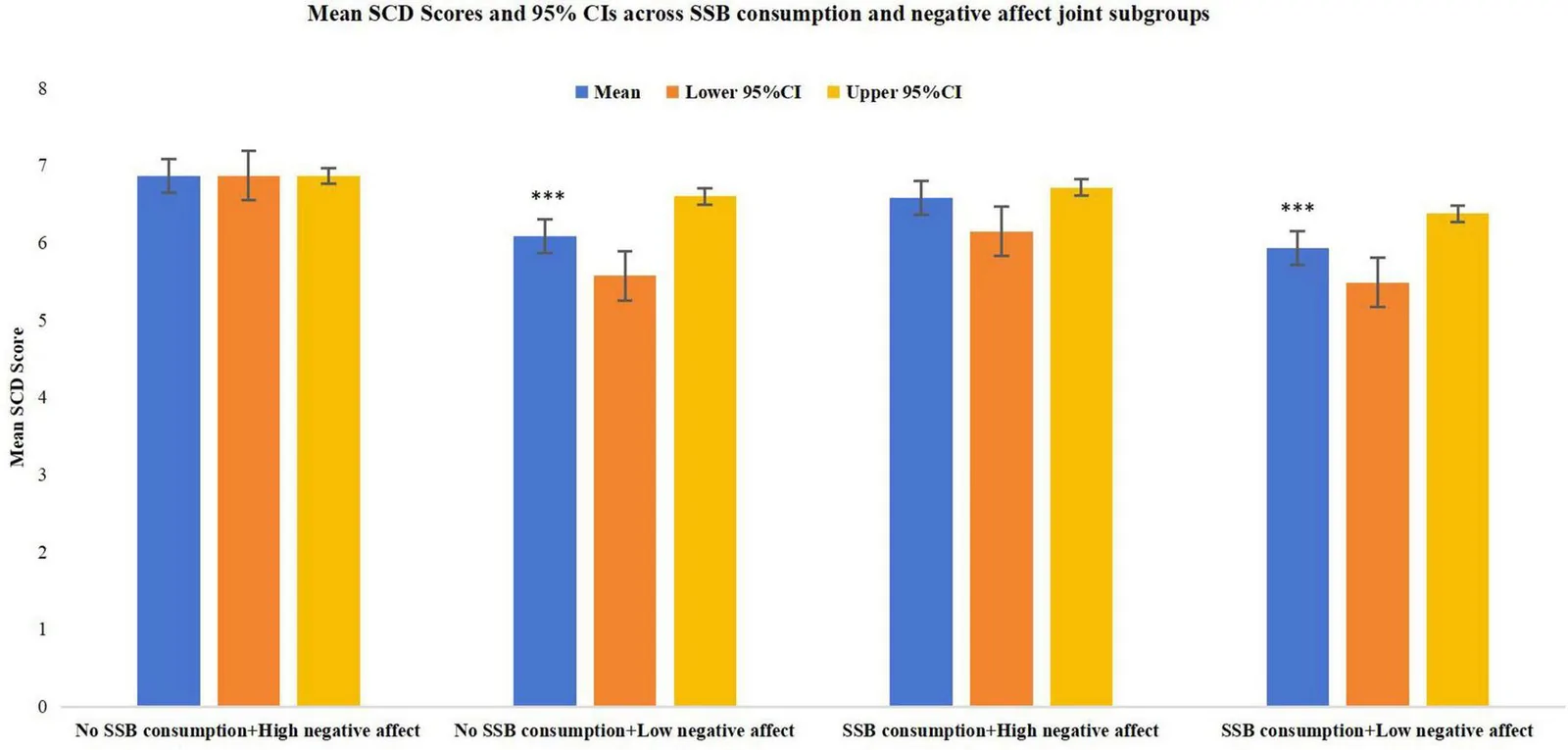
Mean SCD scores across four joint exposure groups of SSB consumption and negative affect among rural older adults. ***Statistically significant intergroup difference.

The results showed that compared with the reference group: Participants in the no SSB consumption + low negative affect group had significantly lower SCD scores (β = −0.778, *P* = 0.003). The SSB consumption + high negative affect group showed no significant difference in SCD scores compared with the reference group (β = −0.283, *P* = 0.199). The SSB consumption + low negative affect group had the lowest SCD scores, with a highly significant reduction compared with the reference group (β = −0.936, *P* < 0.001). The overall model fitted well (*F* = 19.454, *P* < 0.001) (Figure 3 and Table 6).

**TABLE 6:** Joint effect of SSB consumption and negative affect on SCD among rural older adults (*n* = 485).

Variables	β	*SE*	*t*	*P*	95% CI
Constant	6.869	0.468	14.672	<0.001	[5.949∼7.789]
Gender	0.334	0.156	2.136	0.033	[0.027∼0.641]
Education	−0.582	0.141	−4.135	<0.001	[−0.859∼−0.305]
Self-rated health	0.406	0.084	4.810	<0.001	[0.240∼0.572]
No SSB consumption + low negative affect	−0.778	0.261	−2.977	0.003	[−1.291∼−0.264]
SSB consumption + high negative affect	−0.283	0.220	−1.287	0.199	[−0.716∼0.149]
SSB consumption + low negative affect	−0.936	0.227	−4.119	<0.001	[−1.382∼−0.490]
*R*	0.443				
*R* ^2^	0.196				
*F*	19.454				

***Statistically significant intergroup difference.

## 4 Discussion

This study’s key novelties lie in three aspects. First, it systematically examined the interaction between SSB consumption and emotional status. Second, this work simultaneously integrated nutritional and psychological factors associated with SCD. Third, this study investigated these associations within an underrepresented rural older adult population. It should be noted that speculative discussion of potential biological mechanisms is not the primary novel contribution of the present observational study. Collectively, the findings provide preliminary epidemiological evidence and generate hypotheses for future longitudinal and interventional studies.

### 4.1 Influencing factors of SCD among rural older adults

According to demographic factors, female sex showed an independent positive correlation with higher SCD scores, which was in line with Zhang et al.’s findings (22). After controlling for age and educational attainment, Xu et al. (23) discovered that women had a higher prevalence of SCD than men, and that women were more likely to move from SCD to cognitive impairment without dementia (CIND). Ovarian hormone secretion decreases as women go through menopause, increasing the number of women who have cognitive impairment (24). This also explains why cognitive impairment problems are more common in women. Additionally, research has shown that older women with SCD are more likely than their male counterparts to develop dementia (25). These results suggest that more attention should be paid to older women when it comes to managing and preventing SCD in the senior population.

Consistent with previous studies, low education was strongly associated with worse SCD among rural older adults (26). Low educational attainment may exacerbate the adverse effects of white matter hyperintensity burden on cognition (27). These findings are further supported by evidence from the UK Cognitive Function and Ageing Study-Wales, where cognitive reserve was found to attenuate the risk of developing dementia associated with SCD (28). Specifically, higher education may act as a proxy for cognitive reserve, which could help individuals with SCD to compensate for earlier cerebral pathological changes and thus reduce cognitive decline and deterioration (29).

The study discovered that the worse self-rated health correlated with higher SCD scores in rural older adults. According to Taylor et al. (30), people with a history of heart disease, stroke, or chronic obstructive pulmonary disease (COPD) had a considerably higher prevalence of SCD than people without such medical histories. Additionally, Lin et al. (25) discovered that more severe SCD symptoms were strongly linked to prevalent chronic diseases through a cross-sectional study of senior citizens 60 years of age and older in seven communities and two nursing facilities in Guangzhou City. It is clear that SCD among rural older adults is strongly correlated with physical health condition, and that the burden of chronic illnesses plays a major role in exacerbating SCD.

### 4.2 SSB consumption, positive affect, and SCD

A key novel finding of this study was a significant interactive effect between SSB consumption and positive affect with SCD among rural older adults. Simple slope analysis indicated that positive affect was negatively correlated with SCD scores. In contrast, the magnitude of this negative correlation between positive affect and SCD was attenuated for individuals who consumed SSB. Joint subgroup analyses further confirmed this interactive effect. Older adults who did not consume SSB and had high positive affect presented the lowest SCD scores, and the inverse correlation between positive affect and SCD scores was significantly attenuated among SSB consumers. Previous research has separately confirmed that higher positive affect correlates with milder cognitive complaints (31, 32) and that frequent SSB consumption links to poorer cognitive performance in older adults (33, 34), but few studies have integrated both exposures to test their interactive associations, especially within rural older groups in China. This interaction generates hypotheses for future longitudinal or interventional studies and may help identify potentially relevant targets for future research.

Notably, crude unadjusted analyses showed an unexpected trend that SSB consumers presented lower SCD scores. First, residual confounding existed in univariate models. Unadjusted factors including positive affect and unmeasured lifestyle behaviors masked the unfavorable association of SSB consumption. Second, older adults with severe SCD tend to avoid sugary drinks, while those with milder cognitive complaints consume SSB more often (35). After full multivariate adjustment, the interaction of SSB consumption emerged clearly, confirming the counterintuitive crude association was driven by the above biases.

Importantly, SSB consumption was defined as participants with intake ≥ once per month without further refined stratification in this rural community-based survey. We adopted this crude grouping strategy mainly due to the characteristics of the study population: preliminary field investigation indicated that most local elderly residents consumed sugary beverages irregularly, and only a tiny proportion maintained frequent weekly or daily consumption. If we subdivide exposure by consumption frequency, single serving volume, cumulative consumption duration, or beverage subtypes, multiple subgroups would contain extremely sparse sample sizes, leading to unstable regression coefficients and unreliable correlational estimates. The binary grouping guaranteed adequate sample volume in each group to satisfy statistical assumptions for cross-sectional correlational analysis. Nevertheless, this oversimplified exposure measurement inevitably reduces clinical interpretability. By pooling participants with rare monthly consumption and heavy daily consumption into a single SSB consumption group, we cannot capture potential dose-gradient correlational patterns between cumulative sugar load and SCD scores, which may dilute the true magnitude of detected cross-sectional associations. In addition, different categories of sugary beverages differ greatly in added sugar concentration and additive composition; combining all subtypes obscures subtype-specific correlational tendencies relevant to clinical and public health interpretation. Future population-based studies should adopt precise multidimensional exposure assessment protocols: specifically, detailed records of weekly intake frequency, single-serving volume, and cumulative years of consumption, alongside separate categorization of distinct SSB subtypes, to accurately examine dose-response correlational trends and subtype-specific disparities.

### 4.3 SSB consumption, negative affect, and SCD

No significant interactive association between SSB consumption and negative affect was identified in rural older adults with regard to SCD. This indicates that SSB consumption and negative affect may show independent associations with SCD. Existing epidemiological evidence confirms that higher negative affect links to aggravated SCD symptoms among aging populations (36–38). Across four subgroups stratified by SSB consumption and negative affect, high negative affect corresponded to elevated SCD scores regardless of whether participants consumed SSB; the two subgroups with high negative affect presented comparable SCD scores. In contrast, participants with low negative affect showed milder SCD scores, with the lowest SCD scores observed among older adults who consumed SSB yet maintained low negative affect. These subgroup results revealed that low negative affect was consistently linked to milder SCD scores regardless of SSB intake status (39).

The findings provide preliminary epidemiological evidence: lower negative affect was associated with lower SCD scores, suggesting a hypothesis for future intervention research. However, our questionnaire did not collect data covering a full spectrum of potential covariates related to cognitive aging, so we were unable to adjust for a series of unmeasured confounding factors. This incomplete control of confounders may bias the magnitude of the observed correlational relationships among SSB consumption, emotion status and SCD. Subsequent field investigations ought to adopt more comprehensive survey instruments to capture these covariates and achieve adequate adjustment for potential confounders.

### 4.4 Limitations

There were several limitations in this study. Firstly, the current one-time survey cannot establish causal relationships between SSB consumption, positive/negative affect and SCD; only correlational associations can be inferred. Longitudinal follow-up research is required to verify directional effects. Secondly, We categorized SSB consumption as a dichotomous variable ( ≥ 1 time/month vs. none) without stratified analysis by consumption frequency, volume or beverage type. This crude grouping masks potential dose-response relationships. Future population-based studies should adopt precise multidimensional exposure assessment protocols. Thirdly, a small number of participants with actual mild cognitive impairment (MCI) may be included in the SCD group by mistake, which may interfere with the accuracy of the association analysis between SSB consumption, emotion status and SCD. Subsequent studies will combine subjective questionnaires and objective cognitive scales to distinguish pure subjective cognitive decline from mild cognitive impairment. Fourthly, we failed to adjust for multiple well-established geriatric confounders may correlate with SCD, emotion and SSB consumption, including sleep quality, physical activity, smoking, and alcohol use. These unmeasured factors may simultaneously affect exposure, outcome and emotional status, thus distorting the magnitude of observed cross-sectional associations. Future surveys should integrate comprehensive questionnaires to collect the above covariates for sufficient confounding control. Finally, all participants were recruited exclusively from rural Ningxia older populations; findings cannot be extrapolated to urban seniors or residents from other regions of China.

## 5 Conclusion

This study investigated the independent, interactive, and joint effects between SSB consumption, emotional states, and SCD among rural older adults. The main conclusions show that gender, education level and self-rated health were identified as independent factors for SCD scores. Notably, this study detected a significant correlational interaction between SSB consumption and positive affect: the magnitude of the inverse association between positive affect and lower SCD scores was attenuated among older adults who consumed SSB. No significant correlational interaction was observed between negative affect and SSB consumption. Further joint stratified analysis indicated that lower levels of negative affect correlated with milder SCD scores symptoms regardless of SSB consumption, suggesting possible directions that require prospective confirmation. As a cross-sectional analysis, this study cannot establish temporality or causal inference. Longitudinal cohorts and interventional trials are needed to replicate these associations. Overall, the findings provide preliminary epidemiological evidence, generate hypotheses, and may inform future longitudinal and intervention studies.

## Data Availability

The raw data supporting the conclusions of this article will be made available by the authors, without undue reservation.
